# Supplementary material for: Evolution enhances mutational robustness and suppresses the emergence of a new phenotype: A new computational approach for studying evolution
Source: PLoS Comput Biol. 2022 Jan 19;18(1):e1009796. doi: 10.1371/journal.pcbi.1009796 (PMC8803174; doi:10.1371/journal.pcbi.1009796)
Supplement: S1 Fig — The orange and green lines represent the average fitness of each generation, calculated for the lineages obtained by evolutionary simulations, Evo50 and Evo90, respectively. Averages were taken over 100,000 and 55,000 lineages, respectively. The vertical line indicates the fitness at which Ω(f) starts to decrease faster than the exponential rate. (PDF) [file pcbi.1009796.s001.pdf]

S1 Fig

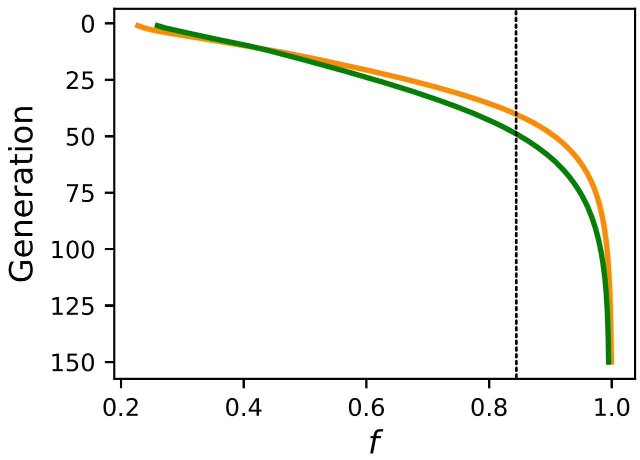

Evolution of fitness for Evo50 and Evo90. The orange and green lines represent the average fitness of each generation calculated for lineages obtained by evolutionary simulations, Evo50 and Evo90, respectively. Averages were taken over 100,000 and 55,000 lineages, respectively. The vertical line indicates the fitness at which $\Omega(f)$ starts to decrease faster than the exponential rate.
