# Supplementary material for: Evolution enhances mutational robustness and suppresses the emergence of a new phenotype: A new computational approach for studying evolution
Source: PLoS Comput Biol. 2022 Jan 19;18(1):e1009796. doi: 10.1371/journal.pcbi.1009796 (PMC8803174; doi:10.1371/journal.pcbi.1009796)
Supplement: S2 Fig — (a) Number of auto-activations nA+. (b) Number of auto-repressions nA−. The data for f ∈ [0.99, 1.0] are shown. The orange solid line and blue dashed line indicate ES and random sampling, respectively. The black dotted line represents the random networks as a reference. The distribution for the ES was corrected using the reweighting method described in the text. Auto-activation is a motif, whereas auto-repression is not a motif. (PDF) [file pcbi.1009796.s002.pdf]

S2 Fig

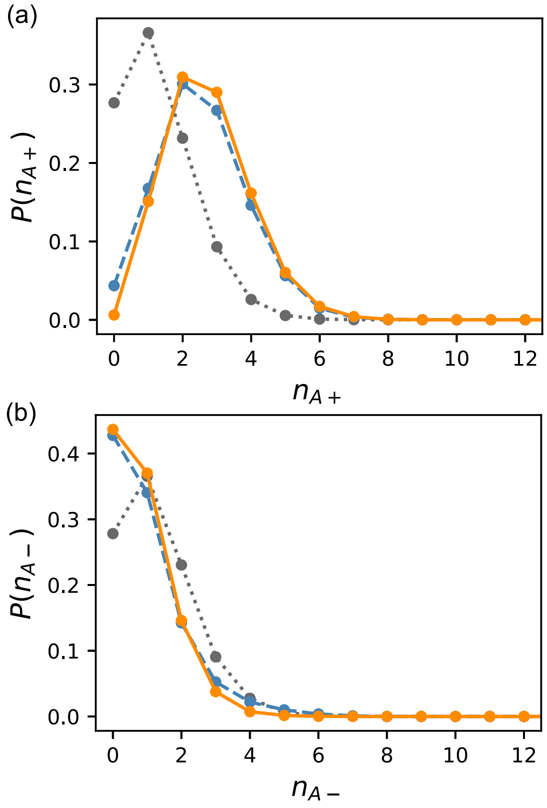

Probability distribution of the number of auto-regulations. (a) Number of auto-activations n_{A+}. (b) Number of auto-repressions n_{A-}. The data for $f \in [0.99, 1.0]$ are shown. The orange solid line and blue dashed line indicate ES and random sampling, respectively. The black dotted line represents the random networks as a reference. The distribution for the ES was corrected using the reweighting method described in the text. Auto-activation is a motif, whereas auto-repression is not a motif.
