# Supplementary material for: Evolution enhances mutational robustness and suppresses the emergence of a new phenotype: A new computational approach for studying evolution
Source: PLoS Comput Biol. 2022 Jan 19;18(1):e1009796. doi: 10.1371/journal.pcbi.1009796 (PMC8803174; doi:10.1371/journal.pcbi.1009796)
Supplement: S3 Fig — The data for f ∈ [0.99, 1.0] are shown. The orange solid lines represent Evo50, and the blue dashed lines represent random sampling. The distributions for the random networks are also shown as references with gray dotted lines. The distributions for the ES were corrected using the reweighting method described in the text. (a) Mutual activation (b) Mutual repression (c) Coherent feed-forward loop (d) Positive feedback loop (e) Mutual activation accompanied by auto-activation of both genes (f) Mutual repression accompanied by auto-activation of both genes. (PDF) [file pcbi.1009796.s003.pdf]

S3 Fig

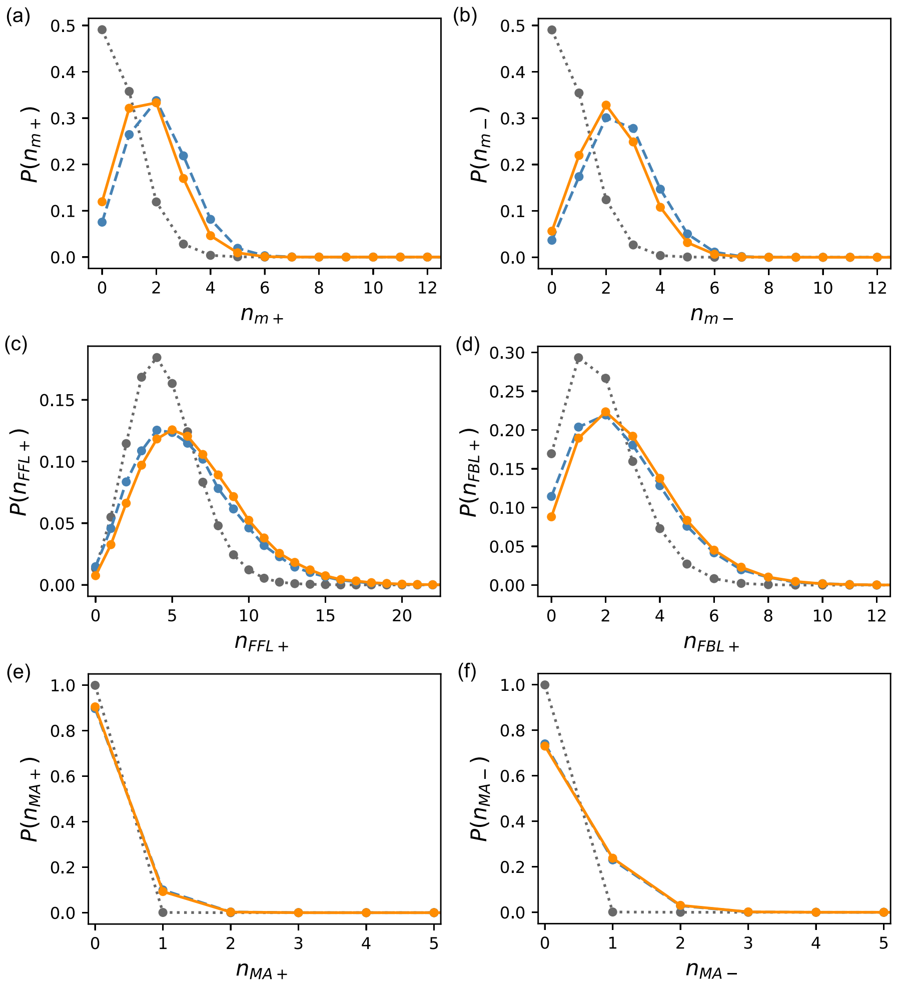

Probability distributions of the number of motifs. The data for $f \in [0.99, 1.0]$ are shown. The orange solid lines represent Evo50, and the blue dashed lines represent random sampling. The distributions for the random networks are also shown as references with the gray dotted lines. The distributions for the evolutionary simulation were corrected using the reweighting method described in the text. (a) Mutual-activation (b) Mutual-repression (c) Coherent feed-forward loop (d) Positive feedback loop (e) Mutual-activation accompanied by auto-activations of both genes (f) Mutual-repression accompanied by auto-activations of both genes.
