# Supplementary material for: Evolution enhances mutational robustness and suppresses the emergence of a new phenotype: A new computational approach for studying evolution
Source: PLoS Comput Biol. 2022 Jan 19;18(1):e1009796. doi: 10.1371/journal.pcbi.1009796 (PMC8803174; doi:10.1371/journal.pcbi.1009796)
Supplement: Attachment — Submitted filename: reply_to_reviewers.pdf [file pcbi.1009796.s004.pdf]

Reply to reviewer 1

The authors consider in silico gene regulatory networks (GRNs) based on discrete time parallel updates of continuous (transcriptional) expression levels along with (i) an associated fitness function and (ii) evolutionary (mutational) dynamics. In that framework they compare properties of GRNs produced within two ensembles: the "uniform" ensemble of all GRNs of a given fitness (referred to random sampling ensemble) and the GRNs resulting from evolutionary dynamics given an initial starting point. The work's main conclusions are as follows. 1: At given fitness, mutational robustness is higher in the second ensemble; 2: the GRNs of high fitness typically exhibit bistability (and thus hysteresis), but less so in the second ensemble, which the authors interpret as a "delayed phenotype" effect; 3: evolution slows down as one reaches high fitness values.

Thank you for carefully reading the manuscript and valuable comments. We largely revised the manuscript considering your suggestions. In particular, as we explain below, we added two new subsections to the "Results" section and reconstructed the "Introduction" and "Summary and Discussion" sections. We largely expanded the discussion. Adding to that, in order to confirm the universality of our results, we conducted additional evolutionary simulations; while in the evolutionary simulation previously made, we preserved the top 50% of GRNs at each generation, in the newly conducted simulation we preserved the top 90% of GRNs at each generation. We presented the results for both simulations in the manuscript. We will call them Evo50 and Evo90, respectively. In what follows, we reply to each of your comments.

1: The author's use of a "random sampling" is both technically a major accomplishment and a useful tool for obtaining insights into what aspects are due to evolutionary dynamics and not just fitness. The authors use a sampling method (a Wang-Landau multicanonical MC) that produces a quantitative characterisation of the "fitness landscape" which allows the authors to refer to what they call "genotypic entropy". That is very good work and I think some of your readers would benefit further if you put a bit more accent on this point.

Considering deeply your comment and another reviewer's comment, we largely change the construction of the manuscript. In the revised manuscript, we stressed to propose the combination of McMC and evolutionary simulation as a new method for studying evolution in general. Then, as applications of the method, we discussed the evolution of the mutational robustness and the emergence of bistability. Accordingly, we no longer put the evolution of the mutational robustness as our central question. We revised largely the "Introduction," "Summary and Discussion," and the "Abstract" in this direction.

We added the following paragraph to "Introduction":

Although evolutionary simulation (ES) is a powerful method for studying the evolution process numerically, its outcomes depend strongly on the evolutionary pathways; therefore, ES alone is insufficient for our purpose. We require a reference system for comparison. In this study, we investigated the evolution of gene regulatory networks (GRNs). A reference system that could be considered natural for this case is a set of all possible GRNs. However, enumeration of all the networks is impossible unless we restrict ourselves to very small GRNs. Thus, the reference system we consider appropriate is a set of randomly sampled GRNs. If some properties are universally observed in that set, they should be realized universally irrespective of the evolutionary pathway. If there are some differences between the results of ES and the reference set, they are manifestations of the particularity of evolution. We propose a research method for determining the properties of the evolutionary process by comparing a randomly sampled set of GRNs obtained using a method based on statistical mechanics with the GRNs obtained using ES. While this method can be applied to studies of evolution in general, we focused on the emergence of bistability and evolution of mutational robustness.

We also added the following paragraph to "Summary and Discussion":

In this paper, we have proposed a new numerical method for studying the properties of evolutionary processes. By generating a reference set via random sampling using the multicanonical ensemble Monte Carlo method and comparing it with the outcomes of evolutionary simulations, we can quantitatively explore the universal properties and particularity of evolution. This method is both powerful and general. We investigated the evolution of a gene regulatory network model.

The authors could have used simulations in the evolutionary ensemble to obtain a characterisation of the same type, leading to genotypic entropy in the evolutionary ensemble too. In physics terminology, you want to answer the question "What is the distribution of fitness in that ensemble", obtainable by direct simulation in the steady state.
Theoreticians would be interested to compare that distribution to the Boltzmann one (adjusting the temperature to get the peak at the same fitness position), as far as I know one has little theoretical control over that distribution. Note that this requested computation uses the steady state distribution in the evolutionary dynamics, distribution that depends on the way mutations are produced and accepted from one generation to the next.
A "noise level" (related to an effective temperature) is simply the fraction of top fitness individuals produced that are passed on to the next generation in your evolutionary simulation; if you accepted more than 500 individuals, the effective temperature would be higher.
I recommend you implement this suggestion so that you no longer use non-equilibrium effects produced by transients and have thus a well defined (steady state) ensemble framework.
Changing to this way of computing in your paper would be little work (in fact the authors perhaps already have the data for the noise level they used) but such a change would significantly enrichen the theoretical and conceptual messages of the paper.
Furthermore I am quite convinced that the 3 main results put forward by the authors would hold in the steady state of the evolutionary dynamics ensemble.
Lastly, note that an advantage of using steady states vs transients is that there then is no dependence on generation number. So to conclude this first point, please do the steady state measurements rather than transient ones and implement the "temperature" parameter in the evolutionary dynamics so that you can scan the average fitness, thereby allowing a much more systematic comparison of the two ensembles.

We conducted additional long evolutionary simulations (2 million generations) by Evo90. As a result, we found that the fitness of all the preserved GRNs reached $f > 0.99999$. But such extremely high fitness should be an artifact of the deterministic dynamics of the GRN model and was not considered to have biological relevance.

Thus, we gave up investigating the steady state by the original model. Instead, we set the upper limit on fitness. Concretely, we regarded the fitness of GRNs greater than 0.99 as f=0.99 and conducted long evolutionary simulations up to 1 million generations. Considering that Evo50 seemed to give rise to a bit too fast evolution, we employed Evo90 for this purpose. The simulations reached the steady state at about 2000 generations. Then we investigated the distribution of lethal edges in the steady state. We obtained the following two results: (1) Fitness of all the preserved GRNs reached 0.99. After that, the evo-

lution became the neutral evolution. Mutational robustness continued to evolve until the steady state was reached. (2) The lethal edge distribution in the steady state of six independent runs converged to only two distinct distributions. This implies that the neutral space is divided into a small number of attracting parts and GRNs in the steady state concentrate in these parts. We described these findings in a new subsection "Steady-state evolution of the mutational robustness" with two new figures (please see the revised manuscript). These results are consistent with the population dynamical theory by van Nimwegen *et al.* (PNAS 17 (1999) 9716), in which the authors discussed the evolution of mutational robustness in the neutral evolution. They are consistent also with the results by Ciliberti *et al.* (PLoS Comput Biol 3 (2007) e15), in which the authors found that the viable GRNs belong to a large cluster in the neutral space. According to our results, either the neutral space of our model is divided into a small number of but more than two parts or the neutral space has a small number of attractors.

We also added the following paragraph in the "Summary and discussions".

> A set of GRNs with high fitness, such as $f \geq 0.99$, compose the neutral space. Thus, we collected the members of the neutral space using our method of random sampling by McMC. Ciliberti *et al.* analyzed the structure of the neutral space for the above-mentioned model in which simple random sampling was valid and found that the high-fitness GRNs belong to a large neutral space [11]. Unfortunately, a similar analysis was difficult for GRNs obtained using McMC. Instead, we set the maximum value $f = 0.99$ and regarded all fitness values greater than it as 0.99 for conducting long ES. After all preserved GRNs reached this maximum value, the neutral evolution continued, and eventually, the steady state was reached. Based on the lethal edge distribution, we conclude that mutational robustness increased during neutral evolution. We found that the populations converged to a small number of distinct distributions of lethal edges. In the present study, the six runs converged to only two distributions. It is natural to assume that GRNs with the same lethal edge distribution belong to the same neutral space or the same attractor in the neutral space. Thus, we consider that the neutral space is divided into only a small number of parts. This finding is consistent with that reported in Ref. [11]. The situation is similar to that studied by population dynamics mentioned above and is consistent with it given $PN\mu \gg 1$ [52]. The population dynamical theory showed that the topology of the neutral space was the main factor in determining mutational robustness. We would like to conduct a detailed analysis of the neutral space for future research.

2: The first of the 3 conclusions given by the authors in their abstract, namely that the second ensemble enhances mutational robustness, is completely expected. Indeed, previous studies have shown that the ensemble based on evolutionary dynamics leads to GRNs with greater mutational robustness than random sampling. That property is very simple to justify qualitatively but it also has a deeper mathematical proof in the case of "0-1" fitness landscapes as given in the paper "Neutral evolution of mutational robustness" (PNAS 1999). So your work can be considered to be a generalization of the results from "0-1" fitness landscapes to arbitrary ones. It is a useful contribution but it is also completely expected. The following analogy from quantum mechanics may help you qualitatively justify the result in your case of continuous fitness landscapes: if one considers a square well potential in 1 dimension, the ground state will have low density at the well's border, corresponding to higher mutational robustness in that ground state compared to the mutational robustness in the trial wave function set to a constant in the well.

As we mentioned above, discussion by van Nimwegen *et al.* (PNAS 17 (1999) 9716) and simulation results by Ciliberti *et al.* (PLoS Comput Biol 3 (2007) e15) showed that the mutational robustness evolves in neutral evolution. Our results of the additional simulation of neutral evolution are consistent with them. Moreover, our results obtained by comparing McMC and evolutionary simulations indicated that mutational robustness and fitness evolves at the same time before the steady state is reached. We consider that this finding is novel, which cannot be obtained from a discussion of the neutral evolution. However, as you pointed out, this can be said an expected result. The novelty of our finding is that we showed it quantitatively by our methodology. Although it may be possible to extend the discussion of van Nimwegen *et al.* to transient evolution, since their theory is for the steady state, its application to the transient state is not straightforward. We added the following paragraphs to "Summary and discussions":

> Evolutionary enhancement of mutational robustness has been discussed for a GRN model with two-valued fitness. Ciliberti *et al.* identified GRNs with a target expression pattern as viable and investigated the effect of natural selection on the viability of mutational robustness [11]. They reported that mutational robustness is enhanced significantly in GRNs that experienced natural selection compared to that of GRNs randomly selected from viable ones. Several different selection pressures have also been reported to lead GRNs to mutationally robust ones [51]. Our results were consistent with these findings. It should be noted that we showed that the enhancement of mutational robustness was observed during the evolution of fitness. Despite the fact that selection is imposed only on fitness, selection of mutationally robust GRNs proceeds with an increase in fitness. We would like to emphasize that mutational ro-

bustness does not evolve after the evolution of fitness ceases, but they evolve simultaneously.

According to the theoretical argument based on population dynamics, when a product of the population size N and mutation rate μ is sufficiently large, mutational robustness is enhanced by neutral evolution [52]. In our case, the evolution was not neutral. However, since mutations that increase fitness are rare, almost all mutations are either deleterious or almost neutral. Thus, evolution is in most part neutral. The deleterious mutations are deleted by selection. In contrast, mutationally robust GRNs are favored in the case of almost neutral mutations. Therefore, while fitness increases intermittently, robustness continues to increase during the period of neutral evolution. In the case of GRNs, the above condition is modified to $PN\mu \gg 1$, where μ is the mutation rate per gene [2]. Because μ is comparatively large in our ES, this condition is considered to be satisfied, and second-order selection works. In this scenario, mutational robustness is enhanced because GRNs containing many lethal edges are easily eliminated from the population. While the above population dynamical theory is for the steady state of evolution, our result was for the transient process of evolution as mentioned above. Thus, the argument above is more or less speculative.

3: The second of the 3 conclusions given by the authors in their abstract, namely that bistability is delayed in the evolutionary ensemble, is an observation based on the authors' simulations but remains unexplained.
Since the authors established it in their "non equilibrium" framework, we don't know if it is due to the transient dynamics or is a feature of the (steady state) evolutionary ensemble. This gives another reason to follow the request I made of using the steady state ensemble in point 1. Probably the result of bistability holds in the steady state ensemble too. Then a possible interpretation and justification for your result would arise if the "bistability" phenotype was anticorrelated with the mutational robustness. (I have to admit that this seems a bit counter intuitive to me but in fact I have very little basis for having any intuition for this subtle point.)
Could you provide a scatter plot and test for such a negative correlation? If the correlation did come out to be negative, you would have a justification for the result, thus a more palatable message.
Whether my intuition is right or wrong, I think the paper would be much improved if you could provide new material to put the claim on more solid ground and include a justification. Without a good result there via a revision, I fear your work will have little impact.

Following your suggestion, we made a scatter plot of fitness and robustness as Fig.9. By classified GRNs into the monostable GRNs and the bistable GRNs, we found that these two exhibited quite different distributions; muta-

tional robustness of the monostable GRNs increases monotonically with fitness. In contrast, the bistable GRNs contain many mutationally fragile GRNs even at high fitness. This finding is non-trivial. This implies that the reason for the delay in the emergence of bistability is that mutationally robust GRNs are favored by evolution. We confirmed that in the similar scatter plot for evolutionary simulations, the mutationally fragile GRNs are substantially reduced at high fitness (results are not shown in the manuscript). To show this result, we added a new subsection "Relation between the bistability and the mutational robustness" in "Results" (please see the revised manuscript). We also added the following paragraph in "Summary and discussions":

> By comparing the outcomes of the evolutionary simulations with random sampling, we clarified that the appearance of bistability is significantly delayed in evolution. At the same time, we found by the random sampling that the bistable group of GRNs contained many mutationally fragile GRNs compared to the monostable group of GRNs. In other words, bistable GRNs and monostable GRNs are intrinsically different in mutational robustness. This is a nontrivial finding, which was revealed by our methodology. From this observation, we conclude that the reason for the delay in the emergence of bistability by evolution is that mutationally robust GRNs are favored in evolution. Nevertheless, as mentioned above, since almost all GRNs become bistable in the high-fitness limit, the evolutionary process should eventually produce a new phenotype, bistability. Thus, bistable and comparatively robust GRNs were selected in evolution. Whether this scenario applies to other phenotypes when different fitness functions are considered is of interest for future studies.

4: The third of the 3 conclusions given by the authors in their abstract, namely that evolution slows down as one reaches high fitness, is in a sense inevitable or even a tautology: if fitness cannot improve its rate of change goes to zero... Nevertheless it is just a qualitative observation whereas in a research paper one expects quantitative measures.
Furthermore, the current approach of the authors which is to consider the dependence of fitness on generation time has 2 major drawbacks: it is based on the outcome of non-equilibrium dynamics and it is logically inevitable since as one approaches the maximum fitness it can no longer increase. If the authors want to keep this point, I recommend they use the same definition of "slowness" as in Monte Carlo via an autocorrelation time.
In the steady state, you can measure that quantity. I would then expect it to grow (slower dynamics) as the selection pressure in the population is increased (or as the mean fitness in the population increases). Allowing such an increase requires that the evolutionary dynamics include a "temperature" parameter, for instance the fraction of kept children over the population size.

As we mentioned above, according to our additional evolutionary simulations, fitness at steady state of evolution becomes extremely high; we consider this is an artifact of the deterministic dynamics of the model and is not biologically relevant. We then tried additional simulations by setting the highest limit of fitness as 0.99 (GRNs with $f > 0.99$ were regarded to $f = 0.99$) and found that all the preserved GRNs at each generation have the highest fitness value, 0.99, and thus fitness does not distribute. Evolution then became neutral. We do not consider that auto-correlation in the neutral evolution is so important; rather we think that the evolutionary speed may be meaningful only in the transient state. We found that Evo50 and Evo90 behaved similarly in the transient state and evolutionary speed slowed down at nearly the same fitness, the result which is shown in S1 Fig. Thus, this finding is universal to some extent. We would like you to admit us limit to a qualitative discussion on evolutionary speed in the transient state in this study.

Minor comments 1: In the introduction, the authors write "Thus, the central question we pose in this study is whether mutational robustness really evolves". Since the stationary probabilities in your reference ensemble and that produced by evolutionary dynamics inevitably are different, there will necessarily be evolution of mutational robustness in your study. I don't think it is good to say your paper is mainly about this point.

Considering your suggestion, we stress to propose a new method to study evolution (a combination of McMC and evolutionary simulation). We consider that a novelty of our finding on mutational robustness is that we showed quantitatively the simultaneous occurrence of the enhancement of the mutational robustness and increase of fitness by evolution. We added the sentences provided in the response to the major point 2 in "Summary and discussions".

2: You write "this response function has a single fixed point even when the auto-activation loop is attached". Please mention that this feature depends on the values of beta and mu (and possibly at which values auto-activation allows for multistability at the single gene level).

The response function we used does not exhibit multistability even if the auto-activation loop is attached as long as $\mu = 0$. An example of parameters that exhibits bistability by auto-activation is $\beta = 6$ and $\mu = 0.5$. We added the following sentences to "Model":

> The response function $R(x)$ with $\mu = 0$ is not ultrasensitive because a single gene with this response function has a single fixed point even when the auto-activation loop is attached. Therefore, for the emergence of multistability, many genes must act cooperatively. An example of the parameters of $R(x)$ that a single gene with an auto-activation loop exhibits bistability is $\beta = 6$ and $\mu = 0.5$.

3: Since the Jij space is discrete and your response function R has a maximum that is strictly less than 1, there has to be a drop in GRN entropy at f approaching that limit which is less than 1. This effect must be very small since it is not visible in Fig 1. But it also means that the two curves in that figure have to become vertical before f=1, close or not to the point where the dependence on f becomes super exponential.

We found it was difficult to generate GRNs with extremely high fitness by McMC. But by very long evolutionary simulations (two million generations), we found that f exceeds 0.9999999; thus, in our model, GRNs exist that have at least this value, which is very close to 1. Thus, the curves do not become vertical until f becomes extremely close to 1, which we could not observe numerically. We added the following paragraph to "Results":

> To investigate the steady state, we conducted very long simulations of Evo90. We found that the fitness of all the preserved GRNs exceeded 0.9999999 at the two-millionth generation. Such extremely high values of fitness should be an artifact of the deterministic nature of GRN dynamics and should not be considered biologically relevant.

4: "In other words, the dynamical system reaches different fixed points for I = 0 and 1." You can improve this sentence so that the readers will understand your point better. The fixed points at I=0 and I=1 are different even at small f. What happens at larger f values is that bistability emerges: in a range of intermediate I values, one has 2 fixed points, having respectively low and high outputs. So although individual genes do not have bistability (for the beta and mu parameters you chose), the GRN does.

We mentioned in the "Result" section that there are two types of GRNs, monostable and bistable, and that their difference is that the latter exhibits the saddle-node bifurcation(s). We added the following sentences:

> In other words, the dynamical system reaches different fixed points for $I = 0$ and 1. When I is changed continuously, two saddle-node bifurcations occur, in contrast to the case of a small f, wherein a single fixed point moves to follow the change in I. We call the latter type of GRNs monostable.

In "Summary and discussions," we further mentioned that even the toggle-switch motif does not give rise to bistability for the parameters we used, and thus the bistability realized as a cooperative phenomenon of many genes. We added the following sentences:

> The response function we used is non-cooperative so that, for example, a well-known toggle-switch motif, that is, the mutual repression of two genes accompanied by auto-activations of both genes, alone

> does not give rise to bistability. Thus, the observed bistability is a cooperative phenomenon of many genes.

5: The high fitness GRNs correspond to amplifying the differential input of node 0. An engineer would be able to design a set of Jijs to do that "simply". Are you able to give such a "design" interpretation to your GRNs of high fitness?

As we mentioned above, the response function we used was not ultra-sensitive and even the motifs that are well-known to exhibit bistability if the response is ultra-sensitive, such as the mutual repression of two genes accompanied by auto-activations of both genes, cannot exhibit bistability. Also, we have not found any characteristic motifs that are pronounced for high-fitness GRNs. Thus, so far, we have not found "design principles" for high fitness; we only interpreted that the high fitness was realized as a cooperative phenomenon of many genes. We would like you to admit we leave this "design principle" problem for future investigations.

6: Out of curiosity, have you understood whether the autoactivation plays any role, i.e. would the results be almost the same without Jii=+1? I assume that all the high fitness GRN have Jii=0 or +1, that is Jii=-1 is "not good" for fitness. Any results there will be welcome.

As you expect, auto-activation is a motif and auto-repression is not a motif for high-fitness GRNs. In other words, $J_{ii} = +1$ is favored compared to $J_{ii} = -1$. But they are not many, and the majority of the diagonal components are $J_{ii} = 0$. In fact, $J_{ii} = +1$ is not indispensable; we found that there are small number of high-fitness GRNs that do not have $J_{ii} = +1$. We showed the number distributions of auto-activation and auto-repression in Fig.7 and added the following sentences:

> In Fig. 7, we compare the number distributions of auto-activation and auto-repression; the former is a motif, while the latter is not. It should be noted that the number of auto-regulations is low. However, there are very few GRNs that do not undergo auto-activation. In contrast, almost half of the GRNs do not have auto-repression. Thus, auto-activation is favored compared to auto-repression but is not indispensable.

7: "This implies that the difference between evolution and random sampling should lie in global network structures." I assume you are comparing the GRNs at a given fitness. Then if it is fitness that drives motifs one does not expect much differences between the two ensembles. The motifs should appear as fitness rises, that is probably the relevant correlation, while the "evolutionary dynamics" ensemble is mainly relevant for mutational robustness.

According to the definition of motifs – structures that are abundant compared to random networks – high-fitness GRNs contain motifs. But they are not pronounced, in contrast to the model in Ref.24 in which abundance of FFL+ and FBL+ motifs were pronounced. Therefore, it seems that motifs do not significantly contribute to high fitness in the present model. We found that the lethal edge distributions are largely different between the monostable GRNs and bistable GRNs. But we consider that local structures such as motifs do not largely affect mutational robustness. We added the following sentences in "Summary and discussions":

> As mentioned above, bistability was not realized by these single motifs but realized as a cooperative phenomenon of many genes. Nevertheless, these motifs were observed in our model, although they were not indispensable. However, they are not relevant to the mutational robustness. This implies that mutational robustness is related to the global structure of GRNs.

8: In the Discussion, you write "The new GRN phenotype, bistability, inevitably emerges as fitness increases". I would be more careful about the claim, although the observation holds in your system, it may not be "inevitable". For instance if the parameter mu were adjustable for each gene, one could probably just use a multi-layer fan out architecture to amplify the deviation from 0.5 of each gene's input signal and there would be no bistability. As a result, I am not so sure that your interpretation of "delayed" phenotypic change is generic. Specifically, I am not sure that mutational robustness (enhanced under evolution) is antagonistic with having a particular phenotype (here multistability). This connects to my point 3 in "Main recommendations of the referee" where I think you can improve your study to really reach a conclusive result.

We agree. Whether or not bistability is inevitable for other parameters should be confirmed by separate studies. But since the same property was observed also in the model in Ref.24, in which the network structures are largely different from our model, this property is to some extent universal. We added the following sentences to "Summary and discussions":

> Nagata and Kikuchi obtained the same results for their GRN model [24]. In contrast to our model, in which any network structures

were allowed, they imposed restrictions on network structure; autoregulation and mutual regulations were prohibited. As a result, the distributions of the network motifs were different for the two models. Nevertheless, both models exhibited similar bistability. Thus, bistability is a universal property for both models, irrespective of the network structure details.

As we mentioned in the reply to major point 3, the reason for the delay in appearance of bistability is the selection by mutational robustness, as is inferred from the scatter plot, Fig.9. The distributions of mutational robustness are markedly different for monostable GRNs and bistable GRNs. This is our new finding. But we would like you to admit we leave the investigation of its deeper reason for future studies.

Reply to reviewer 2

> In this computational study, the authors simulate a simple model of a genetic regulatory network to investigate how the ensemble of genotypes emerging through an evolutionary process is atypical relative to the ensemble of all high-fitness genomes. For this, they capitalize on a method described in a recent paper sharing one of the co-authors, which employed the multicanonical Monte Carlo technique to sample genotypes in a given fitness bin in an evolution-independent way. This approach allows comparing evolved high-fitness genomes to randomly sampled high-fitness genomes. The authors show that the evolved genomes are more mutationally robust than typical.

Thank you for carefully reading the manuscript and valuable comments. We largely revised the manuscript according to your suggestions. In particular, we added two new subsections to the "Results" section and reconstructed the "Introduction" and "Summary and Discussion" sections. We largely expanded the discussion. Adding to that, in order to confirm the universality of our results, we conducted additional evolutionary simulations; while in the evolutionary simulation previously made, we preserved the top 50% of GRNs at each generation, in the newly conducted simulation we preserved the top 90% of GRNs at each generation. We presented the results for both simulations in the manuscript. We will call them Evo50 and Evo90, respectively. In what follows, we reply to each of your comments.

> I would not necessarily call this reported result groundbreaking; it confirms the intuition many people would probably say they have, but it does so in a clear quantitative way, and the technique used here will likely be useful to other studies. (I, for one, was pleased to learn about the capabilities of McMC.) The paper is well-written and, barring a few comments below, clearly explained. I though the introduction could probably do a better job integrating into the context of the existing literature: currently, it focuses rather narrowly on mutational robustness & GRN, but the idea that evolved genomes are atypical in potentially predictable ways recurs in the broader literature, and to me it seems that the authors' approach is relevant to this broader conversation, which might help this work gain broader visibility. Some examples I'm familiar with are Orr, Genetics (2003); Rice Good Desai, Genetics (2015); Tikhonov Kachru Fisher, PNAS (2020); the work of Erik van Nimwegen. I'm not saying these specific references should be added, merely that these papers, or references therein, might inspire the authors to add a broader paragraph to their discussion section, saying where a similar McMC methodology could be useful.

Considering deeply your comment and another reviewer's comment, we largely change the construction of the manuscript. In the revised manuscript, we

stressed to propose the combination of McMC and evolutionary simulation as a new method for studying evolution in general. Then, as applications of the method, we discussed the evolution of the mutational robustness and the emergence of bistability. Accordingly, we no longer put the evolution of the mutational robustness as our central question. We revised largely the "Introduction," "Summary and Discussion," and the "Abstract" in this direction.

We read the suggested papers and other related papers and realized that the work by van Nimwegen *et al.* (PNAS 17 (1999) 9716) is particularly important to our results and we should have included this in the original manuscript. We also found the work by Espinosa-Soto (J Evol Biol 29 (2016) 2321) is related to the present work. We included discussions related to these papers (in particular, van Nimwegen *et al.*) in "Summary and discussions" instead of "Introduction". Although other suggested papers could also be included in the discussion, we would like you to admit we omit them to simplify the discussion.

We discussed these papers and our findings on mutational robustness in "Summary and discussions". As a related work, we discussed also the work by Ciliberti *et al.* (PLoS Comput Biol 3 (2007) e15). van Nimwegen *et al.* and Ciliberti *et al.* discussed the evolution of mutational robustness under the neutral evolution. To investigate the effect of neutral evolution in our model, we conducted additional long evolutionary simulations, in which fitness is limited to 0.99 ($f > 0.99$ are regarded as $f = 0.99$). As a result, after some generations, fitness of all the preserved GRNs at each generation became 0.99, the maximum value, and after that, the neutral evolution continued. We confirmed that the mutational robustness was enhanced during the neutral evolution until the steady state was reached. We found by six independent runs that the lethal edge distributions at the steady state converged to only two distinct distributions. This implies that the neutral space consists of a small number of separate parts and the steady states are attracted to specific parts in the neutral space. We described these results in the new subsection entitled "Steady-state evolution of the mutational robustness". Our findings are consistent with the prediction by van Nimwegen *et al.*, given that the mutation rate is large. Also. they are consistent with the results of Ciliberti *et al.*, in which they found that "viable" GRNs belong to a large neutral network. Our results imply that either the neutral space is divided into small number but more than one parts or the neutral space have a small number of attractors. We added the following paragraphs to the "Summary and discussions":

> Evolutionary enhancement of mutational robustness has been discussed for a GRN model with two-valued fitness. Ciliberti *et al.* identified GRNs with a target expression pattern as viable and investigated the effect of natural selection on the viability of mutational robustness [11]. They reported that mutational robustness is enhanced significantly in GRNs that experienced natural selection compared to that of GRNs randomly selected from viable ones. Several different selection pressures have also been reported to lead GRNs to mutationally robust ones [51]. Our results were consis-

tent with these findings. It should be noted that we showed that the enhancement of mutational robustness was observed during the evolution of fitness. Despite the fact that selection is imposed only on fitness, selection of mutationally robust GRNs proceeds with an increase in fitness. We would like to emphasize that mutational robustness does not evolve after the evolution of fitness ceases, but they evolve simultaneously.

According to the theoretical argument based on population dynamics, when a product of the population size N and mutation rate μ is sufficiently large, mutational robustness is enhanced by neutral evolution [52]. In our case, the evolution was not neutral. However, since mutations that increase fitness are rare, almost all mutations are either deleterious or almost neutral. Thus, evolution is in most part neutral. The deleterious mutations are deleted by selection. In contrast, mutationally robust GRNs are favored in the case of almost neutral mutations. Therefore, while fitness increases intermittently, robustness continues to increase during the period of neutral evolution. In the case of GRNs, the above condition is modified to $PN\mu \gg 1$, where μ is the mutation rate per gene [2]. Because μ is comparatively large in our ES, this condition is considered to be satisfied, and second-order selection works. In this scenario, mutational robustness is enhanced because GRNs containing many lethal edges are easily eliminated from the population. While the above population dynamical theory is for the steady state of evolution, our result was for the transient process of evolution as mentioned above. Thus, the argument above is more or less speculative.

A set of GRNs with high fitness, such as $f \geq 0.99$, compose the neutral space. Thus, we collected the members of the neutral space using our method of random sampling by McMC. Ciliberti *et al.* analyzed the structure of the neutral space for the above-mentioned model in which simple random sampling was valid and found that the high-fitness GRNs belong to a large neutral space [11]. Unfortunately, a similar analysis was difficult for GRNs obtained using McMC. Instead, we set the maximum value $f = 0.99$ and regarded all fitness values greater than it as 0.99 for conducting long ES. After all preserved GRNs reached this maximum value, the neutral evolution continued, and eventually, the steady state was reached. Based on the lethal edge distribution, we conclude that mutational robustness increased during neutral evolution. We found that the populations converged to a small number of distinct distributions of lethal edges. In the present study, the six runs converged to only two distributions. It is natural to assume that GRNs with the same lethal edge distribution belong to the same neutral space or the same attractor in the neutral space. Thus, we consider that the neutral space is divided into only a small number of parts. This finding is

consistent with that reported in Ref. [11]. The situation is similar to that studied by population dynamics mentioned above and is consistent with it given $PN\mu \gg 1$[52]. The population dynamical theory showed that the topology of the neutral space was the main factor in determining mutational robustness. We would like to conduct a detailed analysis of the neutral space for future research.

A few more specific comments:
Major:
1. The "model" section is completely silent on the details of how the evolutionary simulations were performed. Was a single lineage simulated at a time, or a population (how large?) How were mutations implemented? (This is mentioned very late in the paper, and only because it helps explain some finding.) The evolutionary procedure must be clearly described in the methods section.

We described the details of evolutionary simulations in the "Method" section. In each simulation, the population of 1000 random GRNs are prepared and evolutionary simulations are conducted keeping this number of population unchanged. After each run of simulation was over, we picked up the highest-fitness GRN and obtain one lineage it belonged. Since we added new evolutionary simulations, we revised the explanation in the "Method" section as follows:

> We conducted two types of evolutionary simulations: Evo50 and Evo90. For both simulations, we prepared an initial population consisting of 1000 randomly generated GRNs. The population size remained unchanged during the simulations. In each generation of Evo50, we selected the top 500 GRNs according to the level of fitness and made one copy for each. In the case of Evo90, 90% of the population, namely, 900 GRNs, from the highest fitness is selected for preservation, and the remaining 10% of GRNs were discarded in each generation. We randomly selected 100 GRNs from the preserved 900 GRNs and made one copy for each. Then, these copies were subjected to mutation for both simulations. The mutation procedure for each GRN was the same as in the McMC procedure; an edge was deleted randomly from the network, and a new edge was then added between a randomly selected unlinked node pair. As these procedures were repeated, the average fitness of the population increased. After 150 generations for Evo50 and 200 generations for Evo90, we picked the GRN with the highest fitness in the population and traced its ancestors to obtain a single lineage. The population size and number of generations were determined arbitrarily. We repeated this evolutionary simulation 100,000 times and 55,000 times independently for Evo50 and Evo90, respectively, and, as a result, we collected 100,000 lineages and 55,000 lineages, respectively.

For readers' convenience, we added the following sentences to the "Model" section:

> For each ES, we prepared a random population consisting of thousand GRNs. Two types of ES were made: in one type, half of the GRNs in the population were preserved at each generation, and in another type, 90% of GRNs were preserved. We refer to them as Evo50 and Evo90, respectively. In the following sections, we mainly show the results of Evo50, unless otherwise stated. Details of the computational methods are summarized in the Method section.

2. "Evolution behaves conservatively" - I don't know what this means. Evolved genomes are atypical; various observables are skewed in one or another direction, and one can invoke intuitive arguments to rationalize these differences if it helps explain the observations. But interpreting this as "evolution behaves conservatively" / "evolution prevents easy phenotypic changes" does not strike me as justified.

We meant to paraphrase the finding that the emergence of bistability in the evolutionary simulation was delayed compared to McMC. However, since we realized that this expression is confusing, we omitted this sentence.

To investigate the origin of this delay in the emergence of bistability, we made a scatter plot of fitness and robustness in the new Fig.9, which is included in the new subsection entitled "Relation between bistability and mutational robustness" (please see the revised manuscript). We plotted monostable GRNs and bistable GRNs separately and found that they distributed differently. The mutational robustness of monostable GRNs increases monotonically with fitness, whereas bistable GRNs contain many mutationally fragile GRNs even at high fitness. This is a non-trivial finding. This implies that the reason for the delay in the emergence of bistability by evolution is attributed to the fact that mutationally robust GRNs are favored by evolution. We added the following paragraph to the "Summary and discussions":

> By comparing the outcomes of the evolutionary simulations with random sampling, we clarified that the appearance of bistability is significantly delayed in evolution. At the same time, we found by the random sampling that the bistable group of GRNs contained many mutationally fragile GRNs compared to the monostable group of GRNs. In other words, bistable GRNs and monostable GRNs are intrinsically different in mutational robustness. This is a nontrivial finding, which was revealed by our methodology. From this observation, we conclude that the reason for the delay in the emergence of bistability by evolution is that mutationally robust GRNs are favored in evolution. Nevertheless, as mentioned above, since almost all GRNs become bistable in the high-fitness limit, the evolutionary process should eventually produce a new phenotype, bistability.

Thus, bistable and comparatively robust GRNs were selected in evolution.

3. In the concluding paragraph, the authors say that one of their "three main results" is the "relationship between evolutionary speed and genetic entropy". The value of this "genotypic entropy" discussion is unclear to me. As you approach a fitness peak, the further increase slows down. This is neither new nor surprising, and if the authors choose to dedicate a paragraph of the discussion to this (e.g. lines 301-321), I think it should be made more clear what specific point they are trying to make. As it is, the discussion is rather meandering and strangely singles out one particular study by Sato et al.

We computed "genotypic entropy" using McMC, which is a logarithm of the number of possible genotype for each fitness. We consider this is a new result. We agree that the fact that the evolutionary speed slows down significantly near the fitness that the genotypic entropy starts to drop rapidly is not that a striking result, as you pointed out. However, we consider that the novelty of our result is that we showed this quantitatively by computing the genotypic entropy and thus we consider this may be included in our main results. We also conducted a new evolutionary simulations Evo90 and found the similar tendency. Thus this result is to some extent universal and we consider it is meaningful. Following your suggestion, we compressed the discussion on this part. Combined with that the "Summary and discussions" are largely expanded, the mention to Sato *et al.* becomes relatively lighter in the discussion.

4. "If a difference in x_{out} larger than 10^{-6} was observed between these two processes in some range of I, the GRN was regarded as bistable." ¡– The value 10^{-6} seems so incredibly low that I'm wondering if this is a typo. Surely that level of "bistability" would be completely irrelevant for any biological system? I would imagine (or certainly hope) that the findings in the paper should not change if the threshold is set to something more biologically reasonable. Any value here is an arbitrary choice and I do of course understand that this is not meant as a practical model of any real GRN, but still - any value below e.g. 1% seems like a very odd choice. If the findings DO depend on the threshold value, then I would find this concerning and would certainly like to see some discussion of this.

We employed such a strict criterion because bistable GRNs and monostable GRNs are intrinsically different dynamical systems. However, we agree that extremely weak bistability should not have biological relevance. Therefore, we added new data using a looser criterion, in which the threshold of bistability is set as $\bar{x}_{out} > 0.5$. We showed the result in Fig.2(b). Although the fitness at which the rapid increase of bistable GRNs occurred shifted to higher fitness, the overall tendency that the increase of bistable GRNs was delayed in evolution

compared with McMC was unchanged. The same tendency was also observed in additional evolutionary simulations, Evo90. From these results, we consider both the emergence of bistability and its delay by evolution are biologically meaningful and universal phenomena. We added the following sentences to the ”Results” section:

> First, we investigated bistability using a strict criterion. Bistability was checked as follows: Starting from the steady state at $I = 0$, I was increased by 0.001, and the dynamics were run until the steady state was reached. This procedure was repeated for up to $I = 1$. Then, the inverse process, from $I = 1$ to 0, was performed. If a difference in $\bar{x}_{out}$ larger than 10^{-6} was observed between these two processes in a range of I, the GRN was regarded as bistable. We employed such a strict criterion because the monostable GRNs and the bistable GRNs were intrinsically different dynamical systems.
>
> Although the strict criterion of bistability employed above is mathematically meaningful, very weak bistability may be biologically irrelevant, because it may not be distinguishable from monostability in living systems. Thus, we also investigated the bistability using a looser criterion; the checking interval of I was set at 0.01 and the criterion of bistability was set as a difference in $\bar{x}_{out}$ larger than 0.5. The results are shown in Fig 2(b). While the rapid increase in the bistable GRNs moves to a higher f than (a), the tendency that the emergence of bistability is delayed in evolution compared to that of random sampling is unchanged. Therefore, we consider that the delayed emergence of bistability is a biologically relevant phenomenon.

Minor:
Line 113 - I think some reference or mention that this curve was generated by the McMC protocol is necessary also in the text, not just the figure legend.

We added a phrase “obtained by random sampling”.

Line 117 ”GRNs decrease exponentially with f” −¿ number of GRNs?

We change it to “number of GRNs”.

”Fitness landscape is rough” -¿ Is this referring to the wiggles in the curve? A rough/rugged landscape usually means something quite specific, and this doesn’t seem to be the same meaning as here.

Here, we just meant to indicate that the line is jaggy and not more. We changed the expression to “Although the fitness landscape is jaggy”

Line 120 "it was divided into three regions" -¿ unclear what this is referring to

We added the explanation of "three regions" as follows

> it also consists of three regions, namely, the majority around $f \sim 0$, the exponential decrease, and the faster-than exponential decrease,

Are there any error bars in Fig 2? Otherwise how can we tell that it is substantially different?

The standard erros are smaller than the mark. We added the phrase "the standard errors are smaller than the mark" both in the main text and the figure caption.

Line 178: Increased monotonically

We changed "monotonously" to "monotonically".
