# Supplementary material for: Evolution enhances mutational robustness and suppresses the emergence of a new phenotype: A new computational approach for studying evolution
Source: PLoS Comput Biol. 2022 Jan 19;18(1):e1009796. doi: 10.1371/journal.pcbi.1009796 (PMC8803174; doi:10.1371/journal.pcbi.1009796)
Supplement: Attachment — Submitted filename: Review post-revisions.pdf [file pcbi.1009796.s005.pdf]

The authors have revised the manuscript considerably, addressing most of my original concerns but also reframing the message. In the original version, the declared goal was understanding mutational robustness in a model of gene regulatory networks. In the revised version, the intention switched to being more general. I can summarize it as follows: Evolved genotypes are atypical in ways that we can't assess unless we have an (evolution-)independent way of sampling high-fitness examples, and here is a methodology to do so. To illustrate this approach, here the authors use a model of GRNs & investigate properties such as mutational robustness and bistability, but the question and the method are more general.

I applaud this reframing -- I do think this is the broader value of their methodology, and the new angle would do a much better job highlighting the strengths that other researchers would find useful, beyond the narrow context of specifically mutational robustness and/or GRNs. So, the intention is great. But the revised presentation suffers from several serious issues, many of which were actually introduced in the revisions…

In its current form, I am afraid the manuscript is not yet ready for publication / does not yet satisfy the standards of PLoS Comp biology.

Major issue 1: Abstract

Abstract is unclear and in fact reads like faulty logic. Indeed, here are four statements that follow each other (which I am rephrasing for clarity but, I believe, remain faithful to the authors' text):

1. Evolved high-fitness GRNs were depleted for bistable examples, relative to expectation for random sampling of GRNs
2. Evolved high-fitness GRNs was enriched for higher robustness, relative to expectation for random sampling
3. Bistable networks have lower average robustness.
4. The authors conclude (bold emphasis added): "**Therefore**, the delayed emergence of bistability is a **consequence** of the mutation-selection mechanism of evolution."

Problems:

1. "mutation-selection mechanism of evolution" is basically synonymous with "evolution", so a literal interpretation of point 4 is that is simply a repeat of point #2. However, the authors' intended meaning in #4 is to causally link #1 and #2 (that bistability is explained, i.e. CAUSED by selection for robustness). Indeed, this is stated in the last sentence of "significance", and elsewhere in the text. But this brings me to second issue, namely:
2. this reads to me as the fallacy of interpreting correlation as causation. The observations are *consistent* with bistability being caused by selection favoring robustness, but the logic presented in the abstract does not lead to *conclude* this.

Note that the same logic is repeated in the main text (lines 405-412), similarly observing correlation and concluding a causation (emphasis mine).

> By comparing the outcomes of the evolutionary simulations with random sampling, we clarified that the appearance of bistability is significantly delayed in evolution. At the same time, we found by
> the random sampling that the bistable group of GRNs contained many mutationally fragile GRNs compared to the monostable group of GRNs. In other words, bistable GRNs and monostable GRNs are

intrinsically different in mutational robustness. This is a nontrivial finding, which was revealed by our methodology. From this observation, **we conclude that the reason for** the delay in the emergence
of bistability by evolution is that mutationally robust GRNs are favored in evolution.

Continuing with the abstract:

Third, evolution begins to slow down significantly at the fitness level where the number of available GRNs begins to decrease rapidly. This implies that evolutionary speed is determined mainly by "genotypic entropy."

-> Continuing the list of problems:

3. First, I continue to find that listing this rather obvious statement as a key result would raise eyebrows. Conceptually, I agree there is value in explicitly quantifying things, but this requires some comment - what did we gain here from this quantification?
4. Second, the logic of these two sentences is faulty. I'm sure the entropy really IS indeed the main reason for the evolutionary speed decreasing in the simulations, just on general grounds. But the argument in the abstract, again, conflates correlation & causation. The observation that evolution slows down as the # of available GRNs decreases does NOT imply that the decrease of available GRNs is a causal factor; and the word "mainly" is of course especially unjustified.

Note that in the main text, the slow-down is (more correctly) described as "This **may be** because the number of possible destinations that a GRN can transit to by the mutation is restricted by Ω(f)", and later "this result **suggests** that evolutionary speed depends in a large part on genotypic entropy." These statements are more careful and more correct, but are (inadvertently, I'm sure) misrepresented in the abstract.

Major issue 2:

The major reframing introduced so late in the writing process (while the edits were substantial, a large fraction of the text is of course unchanged) made the manuscript enormous and its focus confused. The abstract immediately and exclusively focuses on GRNs; the introduction then insists this is only an example of a more general question, and the central advance is the methodology (comparing evolved objects with unbiased random sampling). But the manuscript then proceeds to a level of detail that can hardly be justified unless characterizing all the intricacies of the robustness in this model is indeed the central aim. (Which it was in the previous version, but in this version the reader is led to believe that this is not the case!) If the aim of the manuscript is to use the robustness analysis to illustrate the general point (about evolved networks being atypical), this message could have been compactly and impactfully summarized in just a few figure panels, as opposed to 9 main text figures.

The same issue is echoed in the discussion. It starts by a neat summary repeating that the objective was to address a general question, using robustness only as an example. But then rest of the long, meandering discussion focuses on what seems to be very model-specific details; many of which seem virtually unrelated to the stated aim of the paper (lines 418-497 - a full page and a half of text!). Again, these details are interesting to discuss in a paper about robustness; but the new paper claims to be about evolution leading to atypical outcomes.

Being well-written is not listed as an explicit requirement for a paper to be acceptable for PLoS Computational Biology. Nevertheless, I cannot wholeheartedly recommend publication of a manuscript that I find much too long and unfocused. I am happy to be overruled here, and if reviewer #1 did not

find this problematic I am willing to retract this objection. But my recommendation would be to enlist a fresh pair of eyes (a reviewer who has not seen the previous version and is not biased by the contrast), as I am not sure a fresh person would find this manuscript understandable.

Minor issues:

Throughout:

The authors often use "largely" when they mean "strongly" or "significantly". The word "largely" is closer in meaning to "mostly", which is often not the intended meaning.

Author summary: "Despite its importance, it has been largely overlooked by non-experts" - I would recommend removing. I think the literature on mutational robustness is substantial…

Line 200-201:

"We employed such a strict criterion because the monostable GRNs and the bistable GRNs were intrinsically different dynamical systems."

This explanation is unclear. What does "intrinsically different" mean? And how does this "intrinsic difference" inform or justify the value for x_out?

Line 211-212

"Substantially low" -> Substantially lower?

Line 213

"This suggests that evolution tends to suppress the emergence of a new phenotype"

-> As phrased, I disagree. This is an *example* of evolution suppressing the emergence of a new phenotype. It shows that evolution *could* do that. It does not in any way suggest that evolution *tends* to do that - that statement is a generalization (to other possible new phenotypes), and such a generalization cannot be "suggested" from observing a single example.

The term "fitness landscape" (line 150 & throughout):

In particular, "fitness landscape is jaggy" (line 160) - I should have been more clear. My concern with this phrase is not just the adjective (the original was: "fitness landscape is rough"). The term "fitness landscape" has a very concrete and very established meaning - namely, the mapping from genotype to fitness, and NOT just the distribution of fitness values. Both "Fitness landscape is rough" AND "fitness landscape is jaggy" clearly evoke the picture of a model where genomes separated by a single mutation can significantly differ in fitness. This is NOT how the authors mean it in this paragraph. The quantity the authors call "fitness landscape" is in fact "fitness distribution", and I would recommend the authors use that term throughout the manuscript - it is equally simple and is more accurate.
