# Supplementary material for: Evolution enhances mutational robustness and suppresses the emergence of a new phenotype: A new computational approach for studying evolution
Source: PLoS Comput Biol. 2022 Jan 19;18(1):e1009796. doi: 10.1371/journal.pcbi.1009796 (PMC8803174; doi:10.1371/journal.pcbi.1009796)
Supplement: Attachment — Submitted filename: reply_to_reviewers.pdf [file pcbi.1009796.s006.pdf]

Reply to reviewer #1

> The authors have worked hard to address the criticisms of both referees and the revised version is definitely of quality.

Thank you very much for your critical reading of the manuscript. We considered your comments and the other reviewers' comments carefully and revised the manuscript accordingly. In the revised manuscript the changes were made mainly on Abstract, the organization of the Results section, and Summary and Discussions. We also changed the title of the manuscript to "Evolution enhances mutational robustness and suppresses the emergence of a new phenotype: A new numerical approach for studying evolution" by adding a subtitle.

The main changes are as follows.

First, we revised the abstract considerably. We declared that the aim of the study was two-fold, that is, a proposal of a new research method and its application to mutational robustness of GRNs. The new abstract starts from the following sentences.

> The aim of this study was two-fold. First, we proposed a new numerical method to investigate the particularities of evolution. Second, we applied this method to a model of gene regulatory networks (GRNs) and explored the evolution of mutational robustness.

We removed the description of evolutionary speed from the abstract because this result is minor, and changed the last part of the abstract as follows:

> We obtained the following results. First, mutational robustness was significantly higher in ES than in the reference ensemble at the same fitness level. In other words, evolution enhances mutational robustness. Second, random sampling revealed that GRNs with high fitness exhibited bistability when a sensitive response to environmental changes was needed. Comparison with ES showed that the emergence of this new phenotype, bistability, was delayed in evolution. Third, the bistable group of GRNs contains many mutationally fragile GRNs compared with those in the non-bistable group. This suggests that the delayed emergence of bistability is a consequence of the mutation-selection mechanism.

Second, we added the following sentences to the last paragraph of the Introduction:

> In this study, we propose a new numerical method that combines McMC and ES to study evolution. Using McMC, we can sample GRNs randomly for each fitness value and thereby construct a reference ensemble to be compared with the results of ES.

Third, we reorganized the Results section. In the new manuscript, we describe the results in the following order:

1. Genotypic entropy and speed of evolution
2. Evolutionary enhancement of mutational robustness
3. Delayed emergence of bistability
4. Relation between bistability and mutational robustness
5. Motif analysis
6. Path distribution
7. Steady-state evolution of the mutational robustness

The subsections from 2 to 4 present our main results and others deal with minor subjects. We think this reorganization made the story of the paper much clearer.

Forth, we revised the Summary and discussion section considerably. The purpose of this revision is two-fold. First, we reorganized the order of paragraphs so that the discussion on the main results are presented earlier. We think this made the flow of the discussion more straightforward. Second, we condensed the discussion and made this section considerably shorter.

We hope that these changes meet your criterion.

> Minor recommendations of the referee
> (1) In your abstract you write "This implies that the delayed emergence of bistability is a consequence of the mutation-selection mechanism of evolution". Although I am happy you were able to confirm my intuition on this issue, it is not a proof. You should replace "implies" in this sentence by "strongly suggests".

We agree that our results did not provide a proof. We changed the sentence as follows:

> This suggests that the delayed emergence of bistability is a consequence of the mutation-selection mechanism.

> (2) The term "universal" in physics is strong and I recommend you remove it and tone down the "universality" claims you make in this paper.

We agree that the term "universal" bears strong meaning. We deleted "universal" or "universality" from the manuscript and replaced them with terms such as "common" and "common properties" and so on.

(3) The term ”randomly sampled GRNs” will be too often interpreted by readers as uniform with no constraint on fitness. I think for your purposes you could replace it by ”GRNs sampled uniformly at given fitness” and possibly refer to this as your ”reference ensemble” of GRNs. (I prefer ”reference ensemble” to ”reference set” as it is more general and is directly taken from statistical physics.

In the last paragraph of the Model section, we added the following sentences to avoid confusion.

> We sampled the GRNs using McMC to construct the reference ensemble. For this purpose, we divided the entire range of fitness into 100 bins. McMC enabled us to sample GRNs such that the numbers of GRNs in all bins were almost the same. The GRNs within each bin were randomly sampled in principle. Hereafter, we call this method “random sampling”.

(4) ”E. Coli” $\rightarrow$ ”E. coli”

We collected ”E. Coli” to ”E. coli”.

(5) You would benefit from having a native English speaker go through the text.

We consulted an English editing service.

(6) I found the ”Summary and discussions” section rather long.

We agree. We condensed that section considerably.

Other comments To follow up on my previous report, perhaps you did not understand my sugestions concerning temperature. Your use of different fractions of the population to duplicate corresponds to different selection pressures. I was implicitly suggesting to use the more standard framework whereby the fitness of an individual gives a probability of reproduction. Generally fitness is written as f = exp(g(genotype)) and the probability to reproduce is f up to a factor depending on the population average of f. This standard method corresponds to Fisher-Wright and allows one to keep the population size constant. My suggestion to introduce a temperature just corresponds to changing that rule to f = exp(g(genotype)/T). A given T produces a steady state ensemble that will be unique, thereby avoiding (at least theoretically) the disconnected components of a neutral network that you find with your approach.

Thank you for a fruitful comments. We misunderstood your former suggestion. We would like to leave this direction of computation for a follow-up study.

Reply to reviewer #2

> The authors have revised the manuscript considerably, addressing most of my original concerns but also reframing the message. In the original version, the declared goal was understanding mutational robustness in a model of gene regulatory networks. In the revised version, the intention switched to being more general. I can summarize it as follows: Evolved genotypes are atypical in ways that we can't assess unless we have an (evolution-)independent way of sampling high-fitness examples, and here is a methodology to do so. To illustrate this approach, here the authors use a model of GRNs & investigate properties such as mutational robustness and bistability, but the question and the method are more general.
> I applaud this reframing – I do think this is the broader value of their methodology, and the new angle would do a much better job highlighting the strengths that other researchers would find useful, beyond the narrow context of specifically mutational robustness and/or GRNs. So, the intention is great. But the revised presentation suffers from several serious issues, many of which were actually introduced in the revisions...
> In its current form, I am afraid the manuscript is not yet ready for publication / does not yet satisfy the standards of PLoS Comp biology.

Thank you very much for your critical reading of the manuscript. We agree that our previous manuscript was rather confusing. As you pointed out, if we focus on our methodology, the manuscript stressed the results on mutational robustness too much. However, we consider that our results for mutational robustness and bistability are also of importance to present in the manuscript. So, in the revised manuscript, we declared that the aim of the study was two-fold, that is, a proposal of a new research method and its application to mutational robustness of GRNs. We hope you kindly admit this change.

We considered your comments and the other reviewers' comments carefully and revised the manuscript accordingly. In the revised manuscript the changes were made mainly on Abstract, the organization of the Results section, and Summary and Discussions. We also changed the title of the manuscript to "Evolution enhances mutational robustness and suppresses the emergence of a new phenotype: A new numerical approach for studying evolution" by adding a subtitle.

The main changes are as follows.

First, we revised the abstract considerably. The new abstract starts from the following sentences.

> The aim of this study was two-fold. First, we proposed a new numerical method to investigate the particularities of evolution. Second, we applied this method to a model of gene regulatory networks (GRNs) and explored the evolution of mutational robustness.

We removed the description of evolutionary speed from the abstract because

this result is minor, and changed the last part of the abstract as follows:

> We obtained the following results. First, mutational robustness was significantly higher in ES than in the reference ensemble at the same fitness level. In other words, evolution enhances mutational robustness. Second, random sampling revealed that GRNs with high fitness exhibited bistability when a sensitive response to environmental changes was needed. Comparison with ES showed that the emergence of this new phenotype, bistability, was delayed in evolution. Third, the bistable group of GRNs contains many mutationally fragile GRNs compared with those in the non-bistable group. This suggests that the delayed emergence of bistability is a consequence of the mutation-selection mechanism.

Second, we added the following sentences to the last paragraph of the Introduction:

> In this study, we propose a new numerical method that combines McMC and ES to study evolution. Using McMC, we can sample GRNs randomly for each fitness value and thereby construct a reference ensemble to be compared with the results of ES.

Third, we reorganized the Results section. In the new manuscript, we describe the results in the following order:

1. Genotypic entropy and speed of evolution
2. Evolutionary enhancement of mutational robustness
3. Delayed emergence of bistability
4. Relation between bistability and mutational robustness
5. Motif analysis
6. Path distribution
7. Steady-state evolution of the mutational robustness

The subsections from 2 to 4 present our main results and others deal with minor subjects. We think this reorganization made the story of the paper much clearer.

Forth, we revised the Summary and discussion section considerably. The purpose of this revision is two-fold. First, we reorganized the order of paragraphs so that the discussion on the main results are presented earlier. We think this made the flow of the discussion more straightforward. Second, we condensed the discussion and made this section considerably shorter.

We hope that these changes meet your criterion.

Major issue 1: Abstract Abstract is unclear and in fact reads like faulty logic. Indeed, here are four statements that follow each other (which I am rephrasing for clarity but, I believe, remain faithful to the authors' text):

1. Evolved high-fitness GRNs were depleted for bistable examples, relative to expectation for random sampling of GRNs
2. Evolved high-fitness GRNs was enriched for higher robustness, relative to expectation for random sampling
3. Bistable networks have lower average robustness.
4. The authors conclude (bold emphasis added): "Therefore, the delayed emergence of bistability is a consequence of the mutation-selection mechanism of evolution."

Problems:

1. "mutation-selection mechanism of evolution" is basically synonymous with "evolution", so a literal interpretation of point 4 is that is simply a repeat of point #2. However, the authors' intended meaning in #4 is to causally link #1 and #2 (that bistability is explained, i.e. CAUSED by selection for robustness). Indeed, this is stated in the last sentence of "significance", and elsewhere in the text. But this brings me to second issue, namely:

2. this reads to me as the fallacy of interpreting correlation as causation. The observations are *consistent* with bistability being caused by selection favoring robustness, but the logic presented in the abstract does not lead to *conclude* this.

Note that the same logic is repeated in the main text (lines 405-412), similarly observing correlation and concluding a causation (emphasis mine).

> By comparing the outcomes of the evolutionary simulations with random sampling, we clarified that the appearance of bistability is significantly delayed in evolution. At the same time, we found by the random sampling that the bistable group of GRNs contained many mutationally fragile GRNs compared to the monostable group of GRNs. In other words, bistable GRNs and monostable GRNs are intrinsically different in mutational robustness. This is a nontrivial finding, which was revealed by our methodology. From this observation, we conclude that the reason for the delay in the emergence of bistability by evolution is that mutationally robust GRNs are favored in evolution.

We agree that our logic was misleading. Our result shows the correlation between the mutational robustness and bistability and we consider this corre-

lation "suggested" that the reason for the delay in the emergence of bistability is mutational robustness. However, as you pointed out, we did not prove this causation explicitly. Thus, we should have used weaker words. Following a suggestion by the other reviewer, we changed "implies" to "suggests" and the corresponding sentence in the abstract is now as follows:

> This suggests that the delayed emergence of bistability is a consequence of the mutation-selection mechanism.

We also changed "mutation-selection mechanism of evolution" to "mutation-selection mechanism." We changed the last sentence of the Author summary similarly as follows:

> Our results suggest that this delay is a consequence of the fact that mutationally robust GRNs are favored by evolution.

We also changed the corresponding sentences in the main text as follows:

> By comparing the outcomes of ES with random sampling, we found that the appearance of bistability was significantly delayed in evolution. Random sampling revealed that the bistable group of GRNs contained many mutationally fragile GRNs compared to those in the monostable group of GRNs. In other words, bistable GRNs and monostable GRNs behave differently in terms of mutational robustness. This is a nontrivial finding that our methodology made possible. This result suggests that the delay in the emergence of bistability may arise from the tendency that mutationally robust GRNs are selected by evolution.

Continuing with the abstract:
Third, evolution begins to slow down significantly at the fitness level where the number of available GRNs begins to decrease rapidly. This implies that evolutionary speed is determined mainly by “genotypic entropy.” → Continuing the list of problems:

3. First, I continue to find that listing this rather obvious statement as a key result would raise eyebrows. Conceptually, I agree there is value in explicitly quantifying things, but this requires some comment - what did we gain here from this quantification?

4. Second, the logic of these two sentences is faulty. I'm sure the entropy really IS indeed the main reason for the evolutionary speed decreasing in the simulations, just on general grounds. But the argument in the abstract, again, conflates correlation & causation. The observation that evolution slows down as the # of available GRNs decreases does NOT imply that the decrease of available GRNs is a causal factor; and the word ”mainly” is of course especially unjustified. Note that in the main text, the slow-down is (more correctly) described as ”This may be because the number of possible destinations that a GRN can transit to by the mutation is restricted by $\Omega(f)$”, and later ”this result suggests that evolutionary speed depends in a large part on genotypic entropy.” These statements are more careful and more correct, but are (inadvertently, I'm sure) misrepresented in the abstract.

We agree that our statement in the abstract on the entropic effect on the evolutionary speed was misleading. We did not prove that the entropy is the main reason. Also, we agree that this is a rather minor result in this work. Thus, we removed the statement on evolutionary speed from the abstract.

Major issue 2:
The major reframing introduced so late in the writing process (while the edits were substantial, a large fraction of the text is of course unchanged) made the manuscript enormous and its focus confused. The abstract immediately and exclusively focuses on GRNs; the introduction then insists this is only an example of a more general question, and the central advance is the methodology (comparing evolved objects with unbiased random sampling). But the manuscript then proceeds to a level of detail that can hardly be justified unless characterizing all the intricacies of the robustness in this model is indeed the central aim. (Which it was in the previous version, but in this version the reader is led to believe that this is not the case!) If the aim of the manuscript is to use the robustness analysis to illustrate the general point (about evolved networks being atypical), this message could have been compactly and impactfully summarized in just a few figure panels, as opposed to 9 main text figures.

As we explained first, we revised the abstract significantly for making the description consistent with the main text. We declared in the abstract and the Introduction section of the main text that both the proposal of a new method and the application to mutational robustness are the aim of this paper . We consider that the results we obtained for mutational robustness and bistability are of value to present in this paper. Thus, we did not shorten the Results section considerably. Instead, we reorganized the Results section to make the story of the manuscript clearer.

Also, we moved one figure (former Fig.7) to supplementary information because the result is minor.

The same issue is echoed in the discussion. It starts by a neat summary repeating that the objective was to address a general question, using robustness only as an example. But then rest of the long, meandering discussion focuses on what seems to be very model-specific details; many of which seem virtually unrelated to the stated aim of the paper (lines 418-497 - a full page and a half of text!). Again, these details are interesting to discuss in a paper about robustness; but the new paper claims to be about evolution leading to atypical outcomes.
Being well-written is not listed as an explicit requirement for a paper to be acceptable for PLoS Computational Biology. Nevertheless, I cannot wholeheartedly recommend publication of a manuscript that I find much too long and unfocused. I am happy to be overruled here, and if reviewer #1 did not find this problematic I am willing to retract this objection. But my recommendation would be to enlist a fresh pair of eyes (a reviewer who has not seen the previous version and is not biased by the contrast), as I am not sure a fresh person would find this manuscript understandable.

As we stated above, we consider that our results for mutational robustness

and bistability are of importance. Thus, in the revised manuscript, we declared that the aim of the paper was two-fold. However, we agree that the Summary and Discussion section was rather too long and badly organized. Thus, we tried to condense the discussion and reorganized the section so that important results were discussed earlier in the section. Accordingly, we revised this section considerably. We hope this revision meets your criterion.

> Minor issues: Throughout: The authors often use ”largely” when they mean ”strongly” or ”significantly”. The word ”largely” is closer in meaning to ”mostly”, which is often not the intended meaning.

We changed ”largely” to more appropriate words.

> Author summary: ”Despite its importance, it has been largely overlooked by non-experts” - I would recommend removing. I think the literature on mutational robustness is substantial...

We removed that sentense.

> Line 200-201: ”We employed such a strict criterion because the monostable GRNs and the bistable GRNs were intrinsically different dynamical systems.” This explanation is unclear. What does ”intrinsically different” mean? And how does this ”intrinsic difference” inform or justify the value for x_out?

Whereas monostable GRNs have only one fixed point throughout the range of input, the bistable GRNs have two fixed points in some range of input. The latter GRNs exhibit two saddle-node bifurcations in response to change in input. Thus, they behave differently as dynamical systems. Although we expressed this difference as ”intrinsic”, we agree that this word was not informative. To clarify the explanation, we changed the sentences as follows:

> We employed such a strict criterion because the monostable GRNs and bistable GRNs were mathematically different as dynamical systems, in that, the number of fixed points was different. Thus, classifying them as strictly as possible is meaningful.

> Line 211-212 ”Substantially low” $\rightarrow$ Substantially lower?

We changed to ”lower”.

Line 213 ”This suggests that evolution tends to suppress the emergence of a new phenotype” → As phrased, I disagree. This is an *example* of evolution suppressing the emergence of a new phenotype. It shows that evolution *could* do that. It does not in any way suggest that evolution *tends* to do that - that statement is a generalization (to other possible new phenotypes), and such a generalization cannot be ”suggested” from observing a single example.

We agree. We removed this sentence.

The term ”fitness landscape” (line 150 & throughout):
In particular, ”fitness landscape is jaggy” (line 160) - I should have been more clear. My concern with this phrase is not just the adjective (the original was: ”fitness landscape is rough”). The term ”fitness landscape” has a very concrete and very established meaning - namely, the mapping from genotype to fitness, and NOT just the distribution of fitness values. Both ”Fitness landscape is rough” AND ”fitness landscape is jaggy” clearly evoke the picture of a model where genomes separated by a single mutation can significantly differ in fitness. This is NOT how the authors mean it in this paragraph. The quantity the authors call ”fitness landscape” is in fact ”fitness distribution”, and I would recommend the authors use that term throughout the manuscript - it is equally simple and is more accurate.

We deleted the term ”fitness landscape” throughout the paper. First, we changed the title of the subsection to ”Genotypic entropy and speed of evolution.” Second, in the main text at the place where the term ”fitness landscape” first appeared in the previous version, we added an explanation of genotypic entropy as follows:

> The blue line in Fig1 (a) shows the base 10 logarithm of the appearance probability $\Omega(f)$ for each bin of fitness f obtained by random sampling. As the logarithm of probability is entropy, we refer it to as the “genotypic entropy.”

In the rest of the manuscript, we used the term ”genotypic entropy” to indicate $\log \Omega(f)$.

Also, we changed line 160 as follows:

> For comparison, we have shown the genotypic entropy for a steeper response function, $\beta = 4$ and $\mu = 0$, in Fig.1(b). It also comprises three regions, namely, the majority around $f \sim 0$, an exponential decrease, and a faster-than-exponential decrease, as in the case of $\beta = 2$ (the jaggy outline is not because of statistical error).
