# Supplementary material for: Evolution enhances mutational robustness and suppresses the emergence of a new phenotype: A new computational approach for studying evolution
Source: PLoS Comput Biol. 2022 Jan 19;18(1):e1009796. doi: 10.1371/journal.pcbi.1009796 (PMC8803174; doi:10.1371/journal.pcbi.1009796)
Supplement: Attachment — Submitted filename: Reply_to_reviewers.pdf [file pcbi.1009796.s007.pdf]

Reply to reviewer #1

In my previous report I no longer had major scientific recommendations. In their revision, the authors handled properly my recommendations and they furthermore improved the logical flow of the paper and streamlined it, taking it from a meadering if not branched and dispersed presentation to a coherent linear demonstration.
I also went through their response to the second referee. Part of his/her reservations overlapped with mine. Since this revision is now readable and follows a clear logic, in my opinion the authors have also responded satisfactorily to the recommendations of this second referee, though clearly he/she had the same reading effort to do as me.

Thank you very much for your effort in carefully reading the manuscript. Your advice was really helpful to improve our manuscript. We considered your additional comments fully and revised the manuscript accordingly.

Minor comments 1: In the title and elsewhere, you could replace ”numerical” by ”computational”.

We replaced ”numerical” by ”computational” both in the title and in the main text.

2: I would not use the term ”lethal edge”: it is the deletion of the edge that is lethal...

We changed ”lethal edge” to ”essential edge”, and added the explanation of essential edge as follows:

> the essential edge means that the deletion of such an edge leads to fitness close to zero.

3: Figure 7 is a bit misleading because the orange points are displayed after the blue ones and so hide them. It might be better to use crosses for blue rather than the circles so that the overlaps are not a problem.

We divided Fig.7 into separate figures (a) and (b) for the monostable GRNs and the bistable GRNs, respectively. Moreover, instead of using the scatter plots, we used the color maps in the revised manuscript, which are more informative than the scatter plots. We changed the description of the figure in the text accordingly as follows:

> Fig.7(a) and (b) show the number distributions of fitness f and robustness r for the GRNs obtained by random sampling for the monostable and bistable GRNs, respectively.

We also changed the caption of Fig.7 accordingly.

4: Although Editage was useful, they did not do as good a job on the abstract and introduction (which is overly ”wordy”). If you could improve the English there, you'll have more readers going to the end of your paper.

We reconsidered Abstracts and Introduction. We condensed them by deleting unimportant or redundant sentences and changed some phrases, considering not to lose the essence. We believe they are now more readable than before. We also changed some sentences in the text for clarity. Please see the highlighted text.

Reply to reviewer #2

I thank the authors for the revisions. They have addressed my concerns. Hopefully the authors find that the changes did indeed benefited clarity.

Thank you very much for your effort in carefully reading the manuscript. Your advice was really helpful to improve our manuscript.
